# Peripheral medulloepithelioma: a rare tumor with a potential target therapy

**DOI:** 10.1186/1479-5876-12-49

**Published:** 2014-02-21

**Authors:** Maria Debora De Pasquale, Maria Antonietta De Ioris, Angela Gallo, Angela Mastronuzzi, Alessandro Crocoli, Raffaele Cozza, Renata Boldrini

**Affiliations:** 1 Department of Pediatric Hematology/Oncology and Stem Cell Transplantation, Bambino Gesù Children’s Hospital, Piazza Sant’Onofrio, 4 - 00165 Rome, Italy; 2 Surgery Department, Bambino Gesù Children’s Hospital, Rome, Italy; 3 Pathology Department, Bambino Gesù Children’s Hospital, Rome, Italy

**Keywords:** Peripheral medulloepithelioma, Treatment, Target therapy, PDGF

## Abstract

**Background:**

Medulloepithelioma (ME) is a rare embryonal tumor predominantly located in the eye or in the central nervous system without an established treatment.

**Case presentation:**

We report of a case of a localized peripheral ME treated with conventional and high dose chemotherapy, surgery and local radiotherapy. At relapse, the tumor tissue revealed a different molecular signature compared to the initial tumor mass. This molecular signature revealed a high expression of platelet derived growth factor receptor (PDGFR). Sorafenib plus irinotecan and temozolomide was started with a 5 month progression free survival.

**Conclusion:**

Our experience suggests a possible role of sorafenib or different PDGFR inhibitors in ME. Targeting treatment could represent an adjuvant and/or alternative therapy for ME and other rare tumors.

## Background

Medulloepithelioma (ME) is a rare embryonal tumor with a distinctive pathology characterized by papillary and tubular patterns recalling the primitive epithelium of the medullary plate and the embryonal neural tube [1].

ME is usually located in the eye or in central nervous system (CNS); a peripheral location has been rarely reported. Intraocular ME is often a well-circumscribed and benign tumor, while CNS ME is an aggressive neoplasm associated with a good prognosis only if completely removed [2,3]. Considering the rarity of peripheral medulloephitelioma (PME), the optimal treatment has yet to be established.

We report a case of PME with an interesting target protein expression suggesting a possible alternative therapeutic strategy for this rare tumor.

## Case presentation

**Figure 1:**
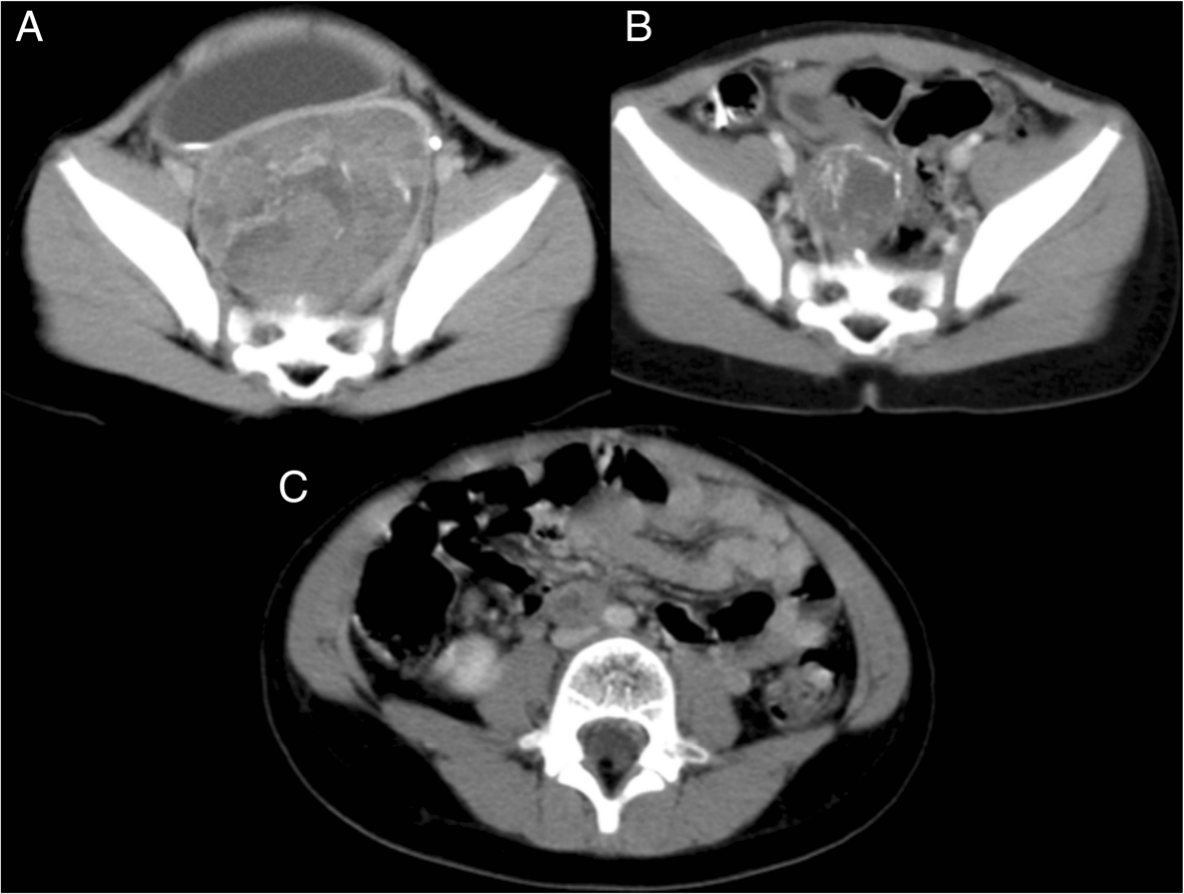
**A: CT scans showing the tumor at diagnosis right hydronephrosis is also present; scans after 4 courses of CT, demonstrating partial response to therapy; C: recurrence of the tumor after six months from stop therapy.** CT at diagnosis (A), after chemotherapy (B) and at relapse (C).

**Figure 2:**
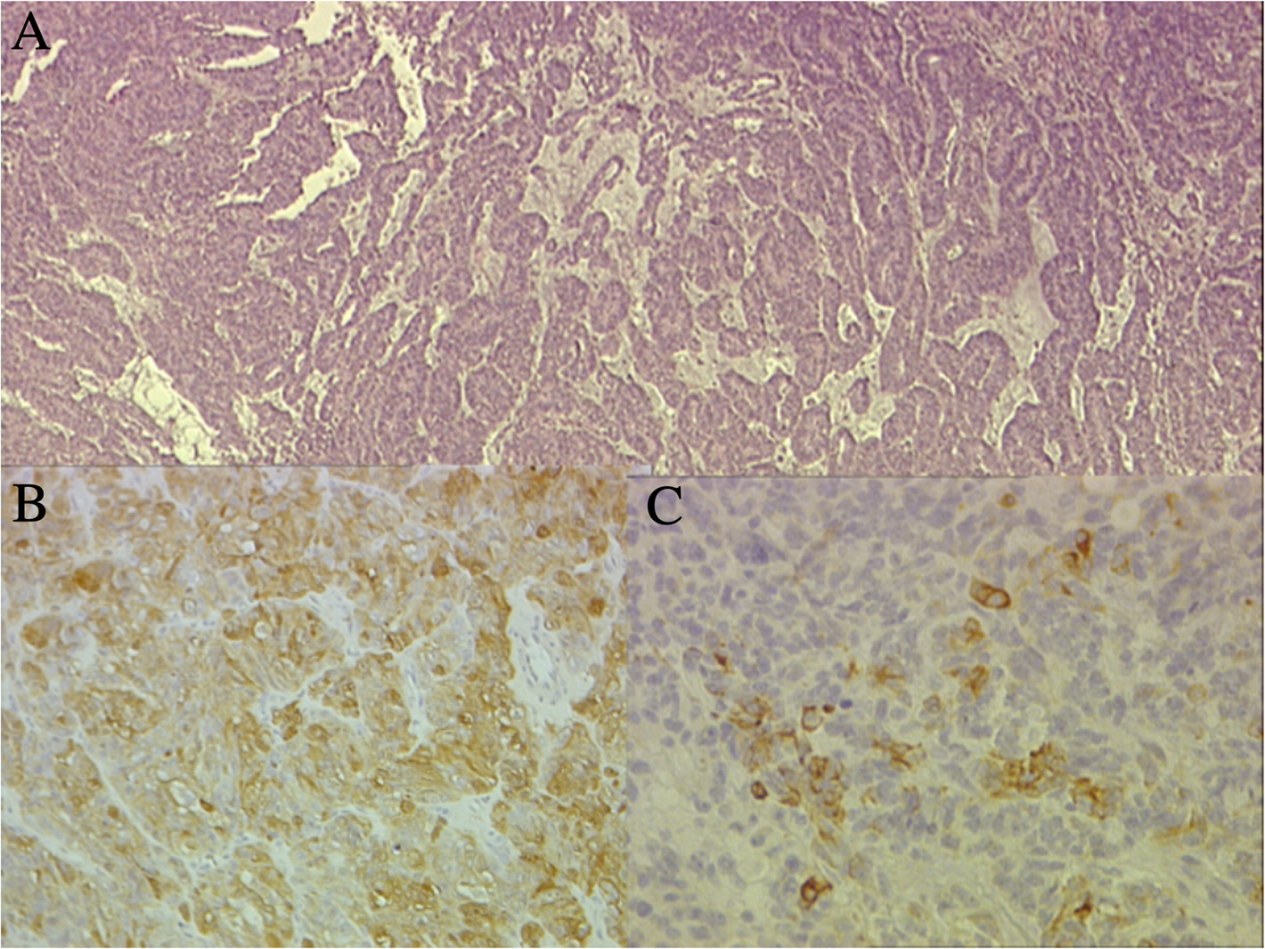
**Pathology of the tumor at diagnosis.** Papillary and tubular patterns represent the distinctive appearance of medulloepithelioma; HE 4 × **(A)**. Positivity of the neoplastic cells for S100 protein 60× **(B)** and for PanCytokeratin 60× **(C)**.

**Figure 3:**
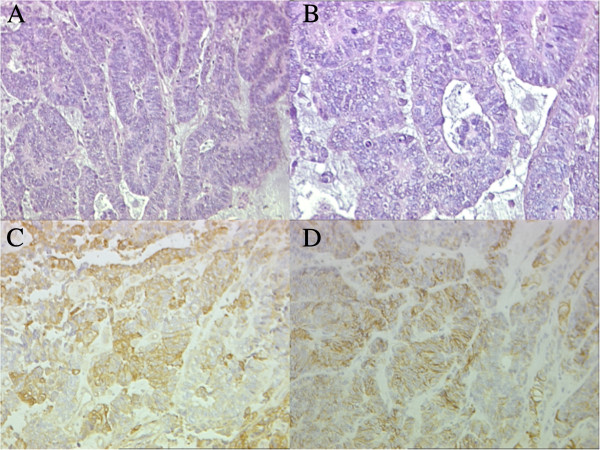
**Pathology of the tumor at diagnosis.** Areas of tumor at higher magnification show stratified columnar cells bordered by internal and external limiting membrane HE 40× **(A)** and 60× **(B)**. Positivity of the neoplastic cells for NSE 60× **(C)** and for CD56 60× **(D)**.

A 3 year old female presented with a one-month history of abdominal pain and anorexia. The abdominal ultrasonography showed a retroperitoneal mass (7 × 6 × 6 cm) confirmed by CT scan with a right *hydronephrosis* without evidence of metastatic spread (Figure 1A). An open biopsy of the lesion was performed. The pathology revealed a malignant neoplasm composed of tubules, papillary structures, ribbons of primitive stratified columnar cells, vesicular nuclei, and high nuclear-cytoplasmic ratio. This histopathology is similar to the structure of the primitive epithelium of the medullary plate and neural tube. The absence of cilia and blepharoplasts ruled out the hypothesis of a papillary ependymoma. The neoplastic cells showed a diffuse positivity for CD56 and WT1 (cytoplasmic) and a variable positivity for NSE, Synaptophisin, S100 protein and Cytokeratin MNF116, while they were negative for CD99, alpha-fetoprotein, CD30, OCT3/4, β-HCG. The diagnosis was neuroectodermal embryonal tumor with patterns of ME (Table 1, Figure 2 and Figure 3).

**Table 1:** Tumor immunochemistry at diagnosis and at relapse

**Immunochemistry**	**Diagnosis**	**Relapse**
CD56	++	++
WT1	++ (cytoplasmatic)	++
NSE	+-	+-
Synaptophisin	+-	+-
S100 protein	+-	+-
Cytokeratin MNF116	+-	+-
CD99	-	-
Alpha-fetoprotein	-	-
CD30	-	-
OCT 3/$	-	-
β-HCG	-	-
EGFR	-	-
PDGFR	+	++
ADAR 1	+	+
ADAR 2	-	-

LEGEND: EGFR, epidermal growth factor receptor; PDGFR, platelet-derived growth factor receptor; ADAR, adenosine deaminases that act on RNA.

The child started chemotherapy according to our local protocol for Ewing Sarcoma Family Tumor [4]. After 2 ICE courses (Ifosfamide 2 gr/m^2^ for 3 days, Carboplatin 400 mg/m^2^ for 2 days and Etoposide 150 mg/m^2^ for 3 days) and 2 CAV (Cyclophosphamide 1.5 mg/m^2^ for 2 days, Vincristine 0.5 mg/m^2^ for 3 days and Doxorubicin 25 mg/m^2^ for 3 days), she achieved partial response, the mass measuring 5 × 3.3 × 3.8 cm (Figure 1B). Grade 4 bone marrow toxicity that required red blood cells and platelets transfusion and hospitalization for neutopenic fever, was recorded after all courses.

**Figure 4:**
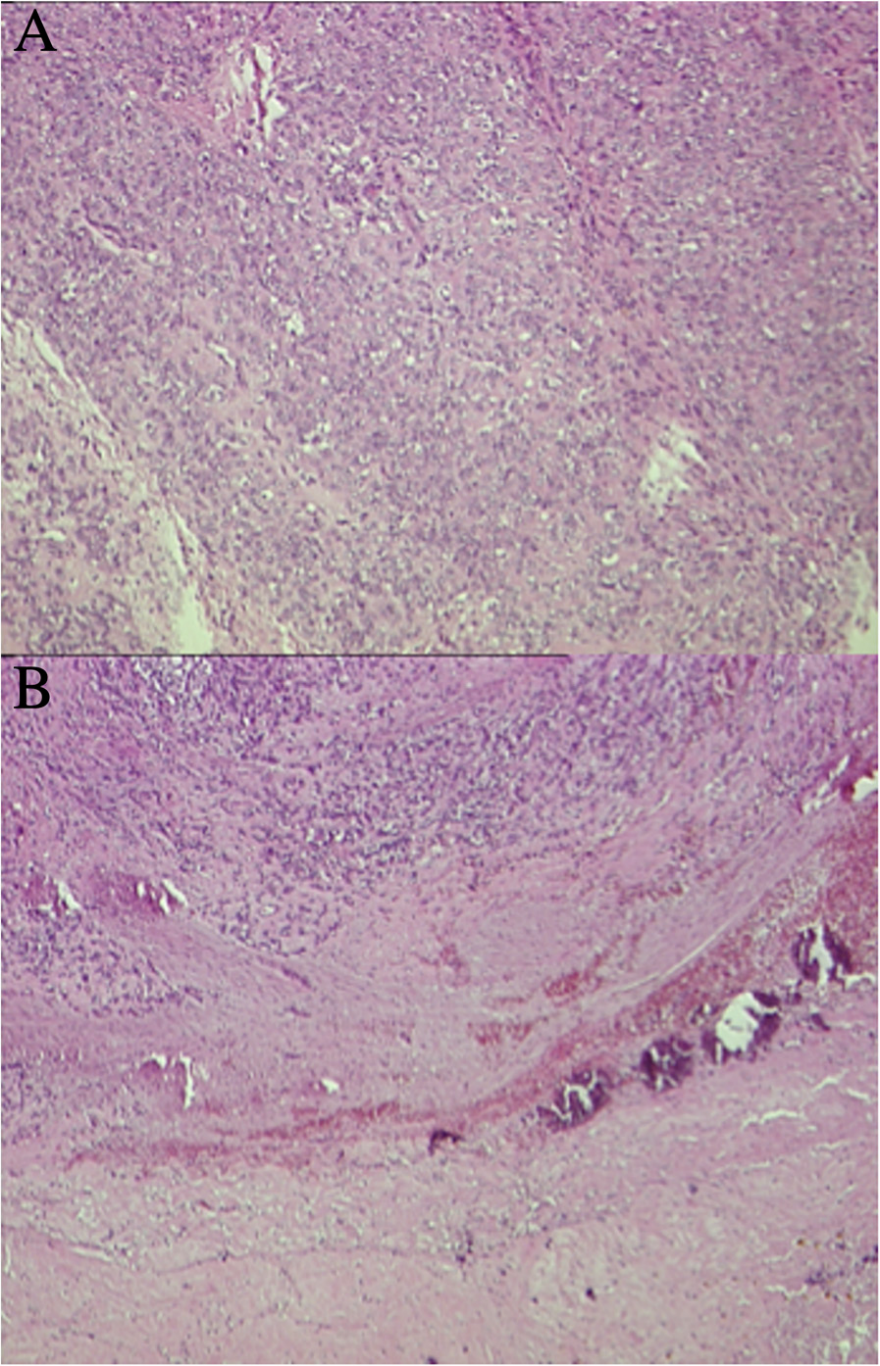
**Pathology after chemotherapy.** The post chemotherapy tumor shows extensive involution area, calcifications HE4 × **(A)** and a predominant solid pattern 4× **(B)**.

A complete resection of the lesion was performed. The pathology showed extensive involutive post-chemotherapy aspects. The residual viable tumor showed histologic aspects overlapping with these of the first biopsy, partly characterized by more solid areas, with the same immunophenotypic pattern (Figure 4).

The child, in complete remission, completed the treatment with two CE courses (Cyclophosphamide 1.5 gr/m^2^ for 2 days and Etoposide 150 mg/m^2^ for 2 days) and a last CAV course. As a consolidation treatment, she received a high-dose chemotherapy based on Busulfan and Melphalan with autologous peripheral blood stem cells rescue and, finally, radiotherapy to the primary tumor bed (19, 5 Gy). During the entire chemotherapy treatment, only grade IV bone marrow toxicity was recorded. During radiotherapy the patient presented only grade I diarrhea.

**Figure 5:**
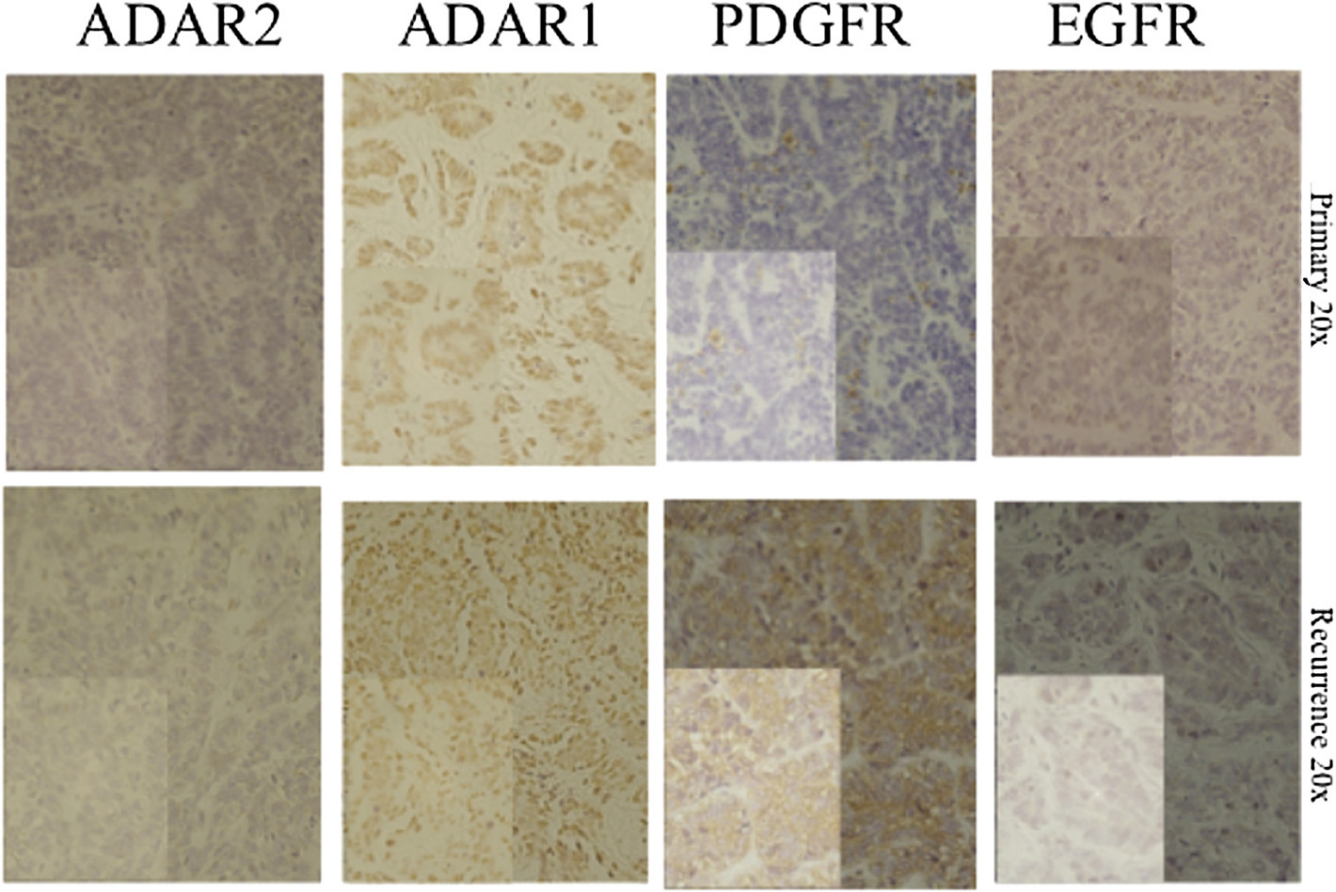
EGFR, PDGFR, ADAR 1 and ADAR2 expression in tumor specimen at diagnosis and at recurrence.

Six months after treatment discontinuation, she presented with an abdominal relapse (Figure 1C). Surgery was performed, achieving a second complete remission. The pathology confirmed a ME with the same characteristics of the primary tumor. At relapse, expression of tumor target proteins was evaluated on tissue specimens obtained both at diagnosis and at recurrence. Immunohistochemistry showed the tumor cells always negative for epidermal growth factor receptor (EGFR). By contrast, there was some positive staining with platelet-derived growth factor receptor (PDGFR) in the primary specimen, with a few cellular clusters showing cytoplasmic positivity, while at recurrence a clear diffuse cytoplasmatic positivity for PDGFR reaching almost 100% of the cells was observed. ADAR2 (adenosine deaminases that act on RNA) was absent in both primary and recurrent tumors; ADAR1 was expressed in the nucleus and cytoplasm, both at diagnosis and at recurrence. Figure 5 shows the EGFR, PDGFR, ADAR 1 and ADAR2 expression in tumor specimen at diagnosis and at recurrence. (See also Table 1).

According to the target protein expression, the patient started sorafenib 200 mg once daily orally, plus temozolomide 100 mg/m^2^/day orally for five consecutive days and irinotecan 10 mg/m^2^/day orally for 14 consecutive days; the course was repeated every 28 days.

Treatment was well tolerated, only generalized skin rash associated with grade I dry skin not requiring treatment discontinuation was recorded. A progression-free survival was achieved for five months when peritoneal carcinomatosis with an important neoplastic peritoneal effusion appeared. The patient died for progressive disease a few weeks later.

## Discussion

PME is a very rare neoplasm and only few reports describe this entity (see Table 2 for more details) [5-9]. Similar to classical ME, surgery in PME, with or without systemic chemotherapy and/or local radiotherapy, represents a therapeutic option. Complete surgery seems to be associated with better outcome [5-9]. All reported PME were located in the pelvic cavity (Table 2). Some authors hypothesized that these tumors originated from undifferentiated cells of the pre-sacral remnant [6]. Four patients with ovarian ME had a good prognosis after surgery either alone or associated with adjuvant chemotherapy and/or radiotherapy [5]. In a case of congenital pelvic ME, prolonged disease-free survival was achieved after partial surgical removal and chemotherapy [7]. In a similar case, pelvic ME was resistant to chemotherapy and the patient died of metastatic pulmonary progression [6].

**Table 2:** Review of literature for peripheral ME

**Reference**	**Sex**	**Age**	**Site**	**Stage**	**Metastasis**	**Therapy**	**Outcome**
Kleinman et al.	F	20 yr	ovary	I	No	Complete surgery, CT	NED, 108 mo
Kleinman et al.	F	32 yr	ovary	I	No	Complete surgery, CT	NED, 36 mo
Kleinman et al.	F	13 yr	ovary	III	No	Complete surgery	DOD, 20 mo
Kleinman et al.	F	23 yr	ovary	III	No	Complete surgery, CT, RT	DOD, 2 mo
Figarella-Branger et al.	F	17 yr	pelvis	IV	Lung	Surgery on primitive tumor, CT	DOD, 8 mo
Bruggers et al.	F	Newborn	pelvis	III	No	Partial resection, CT	NED, 50 mo
Donner et al.	F	12 yr	pelvis	III	No	CT	NED, 36 mo
Nakamura et al.	M	6 mo	pelvis (sciatic nerve)	III	No	Complete surgery	NED, 84 mo

Legend: F, female; M, male; yr, years; mo, months; CT, chemotherapy; RT, radiotherapy; NED, non evidence of disease; DOD, died of disease.

Analyzing the reported cases, it seems that some PME with favorable prognosis were sensitive to chemotherapy, while, for chemo-resistant tumors, the only curative treatment was represented by radical surgery [5-9]. In our case, we decided to treat the patient with an aggressive first-line therapy. The patient achieved complete remission but she relapsed only six months after the end of treatment. At relapse we evaluated the expression of tumor target proteins focusing on the protein expression profile often involved in brain cancers with possible therapeutic implications, such as PDGFR and EGFR. The PDGFR is a cell surface receptor linked to a tyrosine kinase (TK) involved in several processes, including autocrine cancer cells growth and angiogenesis. Few PDGFR antagonists have been developed and introduced in clinical practice, such as sorafenib or STI571/imatinib mesylate [10]. Similarly, EGFR is a cell-surface receptor involved in DNA synthesis and cell migration, adhesion and proliferation. Anticancer drugs directed against EGFR include gefitinib, erlotinib and cetuximab [11].

We also studied the ADARs (adenosine deaminases that act on RNA) expression on tumor specimens. The ADARs are enzymes responsible for Adenosine-to-Inosine conversion on coding and non-coding RNA (such as microRNA) that are emerging as important key proteins in cancers. Recent evidences have connected ADARs (ADAR1 and ADAR2) deregulation to several cancers [12].

Surprisingly, at recurrence, we observed PDGFR expression, not present at diagnosis, in almost all tumor cells.

Sorafenib is a small molecular inhibitor of several TK protein, such as vascular endothelial growth factor receptor, PDGFR and Raf kinases (more avidly C-Raf than B-Raf). The drug has been approved by the U.S. Food and Drug Administration for use in the treatment of advanced renal cancer, unresectable hepatocellular carcinoma and locally recurrent or metastatic, progressive differentiated thyroid carcinoma refractory to radioactive iodine treatment [13-15]. Some authors showed that sorafenib blocks STAT3 (a signal transducer and activator of transcription), as well as the expression of proteins regulating the cell cycle and the apoptosis process, both in cell lines and primary tumor cells of medulloblastoma. These findings provide a rationale for treatment of pediatric medulloblastoma with sorafenib [16]. ME is classified in the group of the embryonal tumors together with medulloblastoma according to WHO classification.

At recurrence, we proposed, as compassionate treatment, sorafenib plus temozolomide and irinotecan. After obtaining IRB approval the three drug combination was started. Treatment was well tolerated and only a mild skin rash was observed.

Sorafenib was chosen according to the PDGFR expression on tumor specimen, while temozolomide and irinotecan had demonstrated activity in medulloblastoma [17,18]. We anticipated an efficacy of this drug combination with a good tolerance and a good quality of life considering the oral assumption.

The finding of poor expression of PDGFR at diagnosis and its massive expression at relapse could suggest that a cell clone with high expression of PDGFR was responsible for the relapse. This leads us to hypothesize that sorafenib, if it had been administered at diagnosis, could have allowed to maintain a longer complete remission.

## Conclusion

Our experience is only a single report, with obvious anecdotal consideration. However this report may suggest that in cases of ME the target protein expression in tumor tissue should be evaluated aimed to identify possible therapeutic targets. Moreover, a possible therapeutic role of PDGFR inhibitors was never reported before.

A TK-inhibitor could be considered and employed also during first line treatment. Indeed, only a multicentric trial could asses the clinical benefit of TK inhibitors combined with chemotherapy; clearly the tumour target protein profile should be evaluated in different phases of treatment as well as at diagnosis or after chemotherapy or at relapse.

Furthermore, we would underline the importance of targets expression study in ME as in all rare pediatric tumors with a poor prognosis in order to identify alternative therapeutic strategies. We also propose regular molecular monitoring of the protein expression profile in tumor samples at reasonable time points during therapy administration. This may help taking decisions in drug selection including appropriate change or adjustment of chemotherapy.

## Abbreviations

ME: Medulloepithelioma; PME: Peripheral Medulloepithelioma; PDGFR: Platelet derived growth factor receptor; CNS: Central nervous system.

## Competing interests

The authors declare that they have no competing interests.

## Authors’ contributions

MDDP conceptualized the report and designed the target studies addressing the patient treatment, collected clinical data, drafted the initial manuscript and approved the final manuscript as submitted. MADI reviewed and revised the manuscript, and approved the final manuscript as submitted. AG performed targeting studies on tumor specimens, critically reviewed the manuscript, and approved the final manuscript as submitted. AM critically reviewed the manuscript and approved the final manuscript as submitted. AC collected clinical data and approved the final manuscript as submitted. RC collected clinical data and approved the final manuscript as submitted. RB designed the target studies, performed and discussed targeting studies on tumor specimens, critically reviewed the manuscript and approved the final manuscript as submitted.
